# Adaptive evidence of mitochondrial genes in Pteromalidae and Eulophidae (Hymenoptera: Chalcidoidea)

**DOI:** 10.1371/journal.pone.0294687

**Published:** 2023-11-21

**Authors:** Ning Kang, Hongying Hu

**Affiliations:** Xinjiang Key Laboratory of Biological Resources and Genetic Engineering, College of Life Science & Technology, Xinjiang University, Xinjiang, P.R.China; Nanjing Agricultural University, CHINA

## Abstract

Pteromalidae and Eulophidae are predominant and abundant taxa within Chalcidoidea (Hymenoptera: Apocrita). These taxa are found in diverse ecosystems, ranging from basin deserts (200 m) to alpine grasslands (4500 m). Mitochondria, cellular powerhouses responsible for energy production via oxidative phosphorylation, are sensitive to various environmental factors such as extreme cold, hypoxia, and intense ultraviolet radiation characteristic of alpine regions. Whether the molecular evolution of mitochondrial genes in these parasitoids corresponds to changes in the energy requirements and alpine environmental adaptations remains unknown. In this study, we performed a comparative analysis of mitochondrial protein-coding genes from 11 alpine species of Pteromalidae and Eulophidae, along with 18 lowland relatives, including 16 newly sequenced species. We further examined the codon usage preferences (RSCU, ENC-GC3s, neutrality, and PR2 bias plot) in these mitochondrial protein-coding sequences and conducted positive selection analysis based on their Bayesian phylogenetic relationships, and identified positive selection sites in the *ATP6*, *ATP8*, *COX1*, *COX3*, and *CYTB* genes, emphasizing the crucial role of mitochondrial gene adaptive evolution in the adaptation of Pteromalidae and Eulophidae to alpine environments. The phylogenetically independent contrast (PIC) analysis results verified the ω ratio of 13 PCGs from Pteromalidae and Eulophidae increased with elevation, and results from generalized linear model confirm that *ATP6*, *ATP8*, *COX3*, and *ND1* are closely correlated with temperature-related environmental factors. This research not only enriched the molecular data of endemic alpine species but also underscores the significance of mitochondrial genes in facilitating the adaptation of these minor parasitoids to plateau habitats.

## Introduction

Within the Chalcidoidea superfamily, Pteromalidae (641 genera and over 4240 species) and Eulophidae (328 genera and over 6054 species) are the two most diverse and populous groups, together accounting for over 10,000 described species [1]. These groups exhibit a broad geographical distribution, thriving in various habitats and spanning wide altitudinal ranges. They also parasitize in the egg or larvae of various agricultural, forestry, and pastoral pests, and have important economic significance [2]. Our previous research indicates that most species of these two families predominantly inhabit lower altitudes, such as basin deserts and grassland, while some of them also thrive in alpine regions like alpine desert and meadow, including *Pachyneuron aphidis* and *Diglyphus isea* [3]. Beyond that, Pteromalidae and Eulophidae indigenous to plateau regions exhibited larger body sizes and superior flight maneuverability, temperature emerged as the dominant environmental factor driving these variations.

Mitochondria, the maternally inherited cells’ powerhouse, are indispensable for maintaining regular life activities and responsible for over 95% of ATP production and energetic metabolism [4]. Typically, the mitochondrial genome consists of 13 protein-coding sequences, 22 tRNA, and 2 rRNA genes. These 13 mitochondrial protein-coding genes (PCGs) encode essential subunits of the electron transport chain (complex Ⅰ, Ⅲ, and Ⅳ) and ATP synthase (complex Ⅴ), which are critical in the oxidative metabolism of carbohydrates and fats, culminating in the generation of water and ATP [4]. The substitution rate of oxidative phosphorylation (OXPHOS) genes in Hymenoptera insects generally exceeds those in other insect orders [5]. Moreover, the Hymenoptera insects exhibit a more pronounced codon usage bias compared to Diptera species [6]. The nucleotide substitution rate of mtDNA considerably exceeds that of nuclear genes [7, 8], making these genes frequently serve as effective tools for species identification, adaptive evolution, and phylogenetic analysis [9–11].

Codon usage bias is a pervasive phenomenon of heterogeneous use of synonymous codons in various organisms. This bias can reflect the critical insights into the evolutionary patterns of genes, enhancing our understanding of gene natural selection and translation efficiency [12]. Factors that influence codon preference include mutation, selection, GC content, random drift, and environmental stress [13]. In this regard, codon bias lends significant depth to our comprehension of genetic evolution driven by environmental stress and identifies the dominant selective forces driving their evolution [14]. To quantify this bias, several indices are frequently employed, encompassing the effective number of codons (ENC or Nc), neutrality plots, the parity rule (PR2) plot analysis, and relative synonymous codon usage (RSCU) analysis.

Parasitoids endemic to the Altun Mountain National Nature Reserve, situated at altitudes beyond 3500 m, consistently encounter substantial environmental stressors including intensified UV exposure, hypoxia, and cold temperatures. Our previous research has verified that alpine species have smaller wing loading than lowland species, indicating that they have stronger flight capabilities. Relevant research indicates that flightless orthoptera accumulate more non-synonymous mutations compared to their flying counterparts [15]. Typically, Hymenoptera exhibiting superior flight abilities correlate with increased consumption of Oxidative Phosphorylation (OXPHOS) [16]. Crucial to OXPHOS, cytochrome c oxidase plays a vital role in enhancing the hypoxia tolerance observed in Tibetan locusts [17]. Additionally, mtDNA PCGs related to metabolism have been identified as adaptive evolutionary targets in flying insects, driven by their heightened energy demands [18]. However, the mtDNA of these minute parasitoids that with greater flight maneuverability at high altitudes has not been verified to be affected by environmental stress.

The important role of mitochondrial genes, which are responsible for energy metabolism in responding to unique alpine environmental factors, is increasingly recognized. Selection pressures within mitochondrial energy metabolism often drive organisms to adjust to the diverse energy requirements in their specific habitats [19]. The gene differentiation and speciation both exhibit an obvious relationship with ambient temperature, while significant evolutionary constraints exist in maintaining and balancing an organism’s energy demands, suggesting that purifying selection could be the predominant force in mitochondrial genome evolution [20]. Numerous studies have confirmed that endemic alpine species exhibit a higher evolutionary rate in mtDNA PCGs. For instance, grasshoppers (Orthoptera) native to Tibetan high-altitude regions exhibit selective pressure signals in five genes (*ATP6*, *ND2*, *ND3*, *ND4*, and *ND5*) [21]. Similarly, grassland caterpillars (Lepidoptera: *Gynaephora*) from the Tibetan Plateau show higher evolutionary rates in PCGs, with *ND5* presenting positive selection signals [22]. A comparative analysis of *Dolycoris baccarum* (Hemiptera) from Tibet and lower altitudes revealed positively selected sites in *ATP6* and *ND5* [23]. The hypoxia and cold-temperature induced stress in harsh environments might be the primary drivers in organismal acclimatization. Thus, the selection pressures on mitochondrial PCGs may drive species to adapt to extreme environment in plateau areas, and also essential for the efficient use of energy.

In this research, we sequenced and annotated the mitogenomes of nine species of Pteromalidae and Eulophidae families, which were collected from alpine grasslands, and seven closely related species from lowland deserts. Coupled with 13 published mitogenomes from the National Center for Biotechnology Information (NCBI) database, we analyzed the characteristics of their 13 PCGs and explored the positive selection sites. We then employed PIC analysis to assess the influence of altitude on evolutionary rate. Additionally, the generalized linear model was conducted to distinguish the relationship between selection signatures and environmental factors. Our study offers fresh perspectives on the adaptive evolution of mitochondrial genes of the Pteromalidae and Eulophidae families and helps elucidates the potential role of mitogenomic evolution in driving the macroevolution of these groups.

## Materials and methods

### Samples, DNA extraction and sequencing

In the present study, we analyzed 29 mitochondrion genomes, 13 of which were previously published and the remaining 16 species were newly sequenced in this study (Table 1). Field specimens were collected from Altun Mountain National Nature Reserve (average altitude: 4500 m) and Junggar Basin (average altitude: 200 m) in Xinjiang Province, China. The fresh samples were collected and preserved in absolute ethanol at -20°C for genomic DNA extraction. The specimens were morphologically identified by Ning Kang using the classic identification systems [24, 25], with voucher specimens deposited in the Insect Collection of the College of Life Science and Technology, Xinjiang University, Urumqi, Xinjiang, China (ICXU).

**Table 1:** Characterization of datasets analyzed in this study.

Gene	Alignment length	Conserved sites	Parsimony—informative sites	Nucleotide composition (%)				Transition/Transversion bias	Best fit model	Pairwise distance
				A	T	C	G			
*ATP6*	777	289	364	36.49	44.87	9.57	9.07	0.44	GTR+G+I	0.23
*ATP8*	291	76	143	42.50	42.74	5.03	4.73	0.69	T92+G+I	0.35
*COX1*	1584	864	577	32.06	43.57	10.93	13.44	0.77	GTR+G+I	0.15
*COX2*	1023	278	365	37.01	42.28	9.30	11.42	0.78	GTR+G+I	0.22
*COX3*	1032	367	413	33.14	45.72	9.47	11.68	0.67	GTR+G+I	0.22
*CYTB*	1191	463	565	34.35	44.34	11.36	9.95	0.73	GTR+G+I	0.20
*ND1*	957	328	531	35.58	46.0	7.19	11.22	0.50	GTR+G+I	0.24
*ND2*	1209	220	820	38.53	48.95	5.94	6.58	0.85	GTR+G	0.38
*ND3*	504	163	207	37.38	47.28	6.77	8.56	0.80	TN93+G	0.45
*ND4*	1530	392	869	36.49	47.56	6.19	9.77	0.48	GTR+G+I	0.37
*ND4L*	438	93	192	37.18	50.91	3.78	8.12	0.71	T92+G	0.27
*ND5*	1818	528	1049	37.14	47.40	6.43	9.03	0.49	GTR+G+I	0.27
*ND6*	762	144	456	41.73	46.53	7.30	4.44	0.69	GTR+G+I	0.33
Concatenated sequence	10,995	3544	6253	35.81	46.15	8.18	9.87	0.55	GTR+G+I	0.24

We extracted total genomic DNA from individual specimens using the TIANamp Genomic DNA Kit (TIANGEN, Beijing, China). The purity and concentration of each DNA sample were detected through agarose gel electrophoresis. Specifically, we mixed 2–5 μl of DNA lysis solution with 0.4 μl of 6× loading buffer and detected using a 0.75% agarose gel (containing 0.5 ug/ml EB). Samples were then sent for DNA sequencing by NovaSeq 6000 (Berrygenomics, Beijing) using pair-end reads with 150 bp length, generating 7-8G raw data and filtered, then assembled and annotated using NOVOPLasty v.4.3 [26] and MITOZ v.2.4 [27] based on the published mitogenomes of Pteromalidae and Eulophidae (NC_058228 *Anisopteromalus calandrae*; MT712142 *Pachycrepoideus vindemmiae*; NC_039656 *Pteromalus puparum* et al.), then manually adjusted the start and stop codons’ position of PCGs, all the results of the annotations were compared and validated with related species using Blast. tRNA genes were identified using tRNA-Scan SE v.1.3.1 [28]. The newly sequenced mitogenomes were deposited in the Genbank (S1 Table in S1 File).

### The comparative analysis of mitochondrial protein coding sequences

CodonW v.1.4.4 was used to analyze the relative synonymous codon usage (RSCU), the effective number of codons (ENC), and the GC content of codons at the 3rd position (GC3s) of the mtDNA PCGs. The skew values of nucleotide composition were calculated using the following formulas: AT skew = (A-T)/(A+T), GC skew = (G-C)/(G+C). The RSCU indicates the ratio between the actual frequency of a specific codon and the abnormal frequency, which is used to detect differences in codon usage among genes. The ratio of ENC and GC3s was used to investigate the main factors causing codon usage bias. If the ENC value is far below the fitted curve, natural selection plays a dominant role in the formation of codon bias. The Neutrality plot (GC12 Vs CG3) is an effective method to investigate the cause of codon bias. When the slope value of the regression line is 1, codon usage bias is completely influenced by mutation, when the slope is close to 0, selection is the primary factor for codon bias. The relative synonymous codon usage among the mitogenomes of 29 species was represented with heatmaps. Data visualization of other relevant datasets was performed with ggplot2, ggpubr, and ggpmisc in R v.4.2.3.

The number of synonymous and nonsynonymous substitutions, along with nucleotide diversity for 13 PCGs, was calculated in DnaSP v.6.0 [29]. We evaluated neutral evolution using the concatenated sequence of all 13 PCGs by conducting a linear regression in PAST v.4.12 [30]. This analysis examined the relationships between the total number of mutations and the base length; the number of synonymous mutations and the sequence length; the number of nonsynonymous mutations and sequence length; the number of synonymous mutations and the number of nonsynonymous mutations.

### Phylogenetic analysis

We performed a phylogenetic analysis of 29 species based on their 13 mtDNA PCGs (*ATP6*, *ATP8*, *COX1*, *COX2*, *COX3*, *ND1*, *ND2*, *ND3*, *ND4L*, *ND4*, *ND5*, *ND6*, *CYTB*). Among them, 11 species are endemic to alpine regions and 18 inhabit lowland areas. A species from the Torymidae (MG923516.1 *Torymus* sp.) was selected as an outgroup in our analysis.

We concatenated all 13 genes of each species and then aligned the sequences with MAFFT v.7.505 [31] using default parameters. We used DAMBE v.7.3 [32] to analyze base substitution saturation. After optimizing the alignment sequences with Gblocks v.0.91 and trimAI v.1.2b [33], calculating the best evolutionary model (GTR+F+I+G4) with ModelFinder v.3.7 [34] then constructed the BI (Bayes inference) tree with Mrbayes v.3.1.2 [35] using 4 Markov Chain Monte Carlo (MCMC) chains, running for 2 million cycles and sampled every 1000 generations. We used the Interactive Tree of Life (ITOL) website [https://itol.embl.de/] to visualize and optimize the BI tree.

### Detection of positive selection site

The ω values of the terminal branches were calculated as an indicator to evaluate the selection pressure. The ratio of non-synonymous to synonymous substitutions (ω = dn/ds) of 13 mtDNA PCGs was estimated using the codon-based method with the CodeML in PAML v.4.9j [36]. We used the free ratio model to calculate evolution values for each branch. The branch models test detects whether selective pressure exists on particular branches, the two-ratio model allows a background ω ratio and a different ω ratio for foreground branches of interest. We used it to compare and analyze the difference in ω values between alpine and lowland populations. In our research, alpine branches were coded 1 as foreground lineages and the lowland species were the background branches, likelihood ratio tests between one-ratio and two-ratio trees were conducted to estimate the significant ω differences between selected branches and other branches.

To further quantify the probability of positive selection at each site in all sequences, we ran the site models (M1a and M2a, M7 and M8). The branch-site model was conducted to analyze positive selection sites along specific lineages. For all the models, codons with positive selection were analyzed using the Bayes Empirical Bayes (BEB) method and sites presented posterior probability (pp)>0.95 were regarded as candidates for positive selection. To verify the presence of positively selected genes, we used MEME (Mixed Effects Model of Evolution) [37], FEL (Fixed Effects Likelihood) [38], FUBAR (Fast, Unconstrained Bayesian Approximation) [39], and SLAC (Single-Likelihood Ancestor Counting) [38], implemented in the Hyphy, to identify codons under positive selection. Sites with significance <0.1 (FEL and SLAC), posterior probability >0.9 (FUBAR), and p<0.05 (MEME) were considered candidates for positive selection. After each model was completed, we examined the substitution rate of each site under positive selection to avoid false positives as much as possible.

### Phylogenetic independent contrast and environmental factor analysis

Closely related species often share similar genetic traits, which may affect the comparative analysis of species. Consequently, we investigated the relationship between altitude and ω values of the 13 mtDNA PCGs using PIC analysis [40], employing the ape package implemented in R v.4.2.3. We used Figtree to convert the Bayesian tree into a binary tree. The binary tree file served as the input file for the analysis. We categorized the 29 species into two groups: alpine group (coded as 1) and lowland group (coded as 0). The ω values were calculated from the selection pressure analysis.

We employed a generalized linear model (GLM) to analyze the relationship between ω values and the environmental factor matrix. We extracted 19 bioclimatic variables from WorldClim 2.1 [41] for 16 field-collected specimens using ArcMap 10.6 and converted the data to a Euclidean distance matrix using the vegan package in R v.4.2.3. The Mantel test was performed to analyze the correlation between two matrices, followed by stepwise regression to screen out the colinear variables, and then conducted the GLM using the Ime4 package.

## Results

### Mitogenome characterization and codon usage comparative analysis

In our research, the newly sequenced mitogenome size varied between 13,193 bp (*Diglyphus poppoea*) and 16,538 bp (*Halticoptera moczari*) (S1 Table in S1 File). All the species contained 13 PCGs and two rRNA genes, trnM, V, A, Q, I was missing in several species except the complete mitogenome of *Sphegigaster intersita* (16,151 bp). The 13 PCGs that all species shared were using ATN as the initiation codon and ended up with complete stop codons (TAA or TAG) or truncated (TA or T). Characteristics of the 13 PCGs were similar to other published species. The AT content of those complete mitogenome sequences ranged from 75.4% (*Diglyphus begni*) to 90.6% (*Selderma saurus*) and the AT content of 13 PCGs ranged from *COX1* (75.6±1.25%) to *ATP8* (90.29±2.37%) (Table 1, S1 Fig in S1 File), the higher AT content brings certain difficulties in complete sequencing. We then mainly focused on the comparative analysis of 13 PCGs that exhibit natural selective pressure in alpine cold and hypoxic environments.

**Fig 1:**
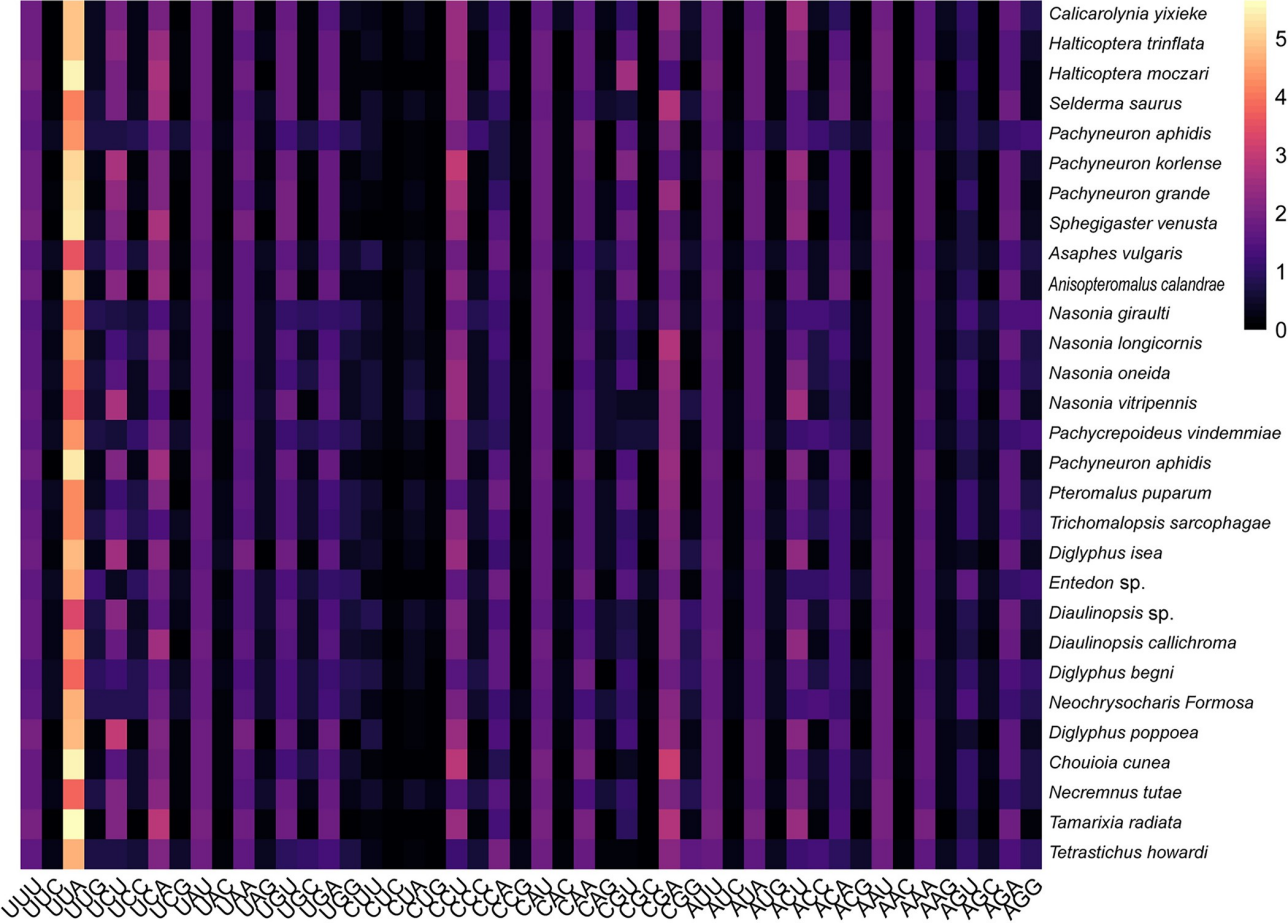
Heat map based on the relative codon usage of the 29 species analyzed in this study.

According to the codon usage analysis results, eight codon families (Ser, Leu, Ala, Arg, Gly, Pro, Thr, Val) exhibit a significant preference for all the species. Codon usage heatmap indicates that UUA(4.552), CCU(2.156), CGA(2.207), GCU(2.114), and UCA(2.079) were the most frequently used codons in the analyzed coding genes (Fig 1), the most frequently used codons were ended with A or T, which is in agreement with previous researches [6]. The AT-skew (-0.171 to -0.084) for 13 PCGs was obviously negative and the GC-skew (0.018 to 0.162) was positive, which indicates that T with a significantly higher occurrence than A, while G with higher occurrence than C.

**Fig 2:**
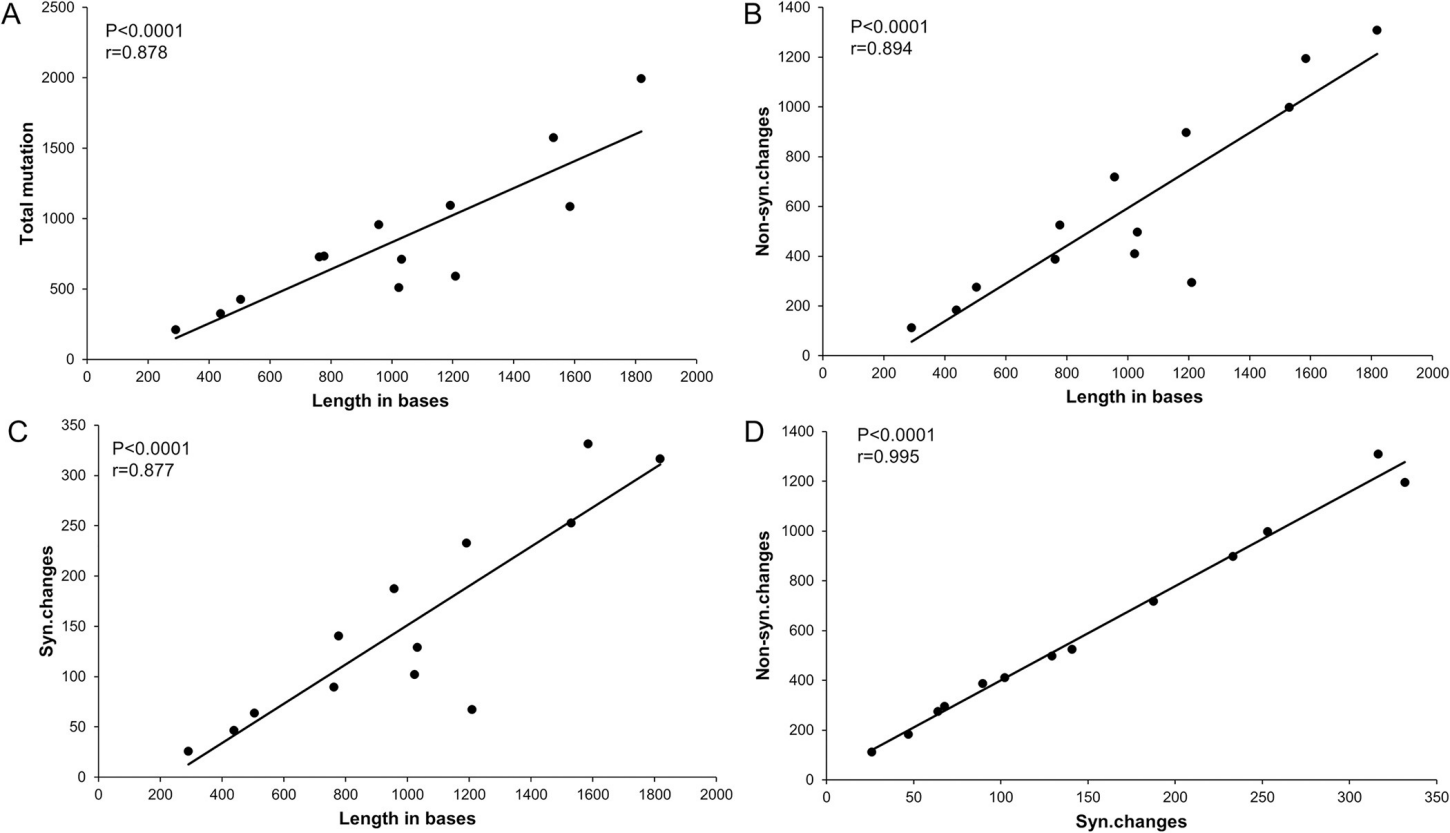
Correlation analysis. Each dots indicate the individual values for 13 PCGs in Pteromalidae and Eulophidae, and the line is the best-fitted line. (A) Correlation between the number of total mutations and length in bases. (B) Correlation between the number of nonsynonymous mutations and length in bases; (C) Correlation between the number of synonymous mutations and length in bases; (D) Correlation between the number of synonymous mutations and nonsynonymous mutations.

Sequence length, total, synonymous, and nonsynonymous mutations were all normally distributed and exhibited a significant linear correlation (Fig 2), the ratio of nonsynonymous mutations was higher in *COX1* (75.46%), *CYTB* (75.39%), and *ND1*(75.07%), while *COX2* (50%) and *ND2* (48.9%) have the fewest mutation sites, relatively.

**Fig 3:**
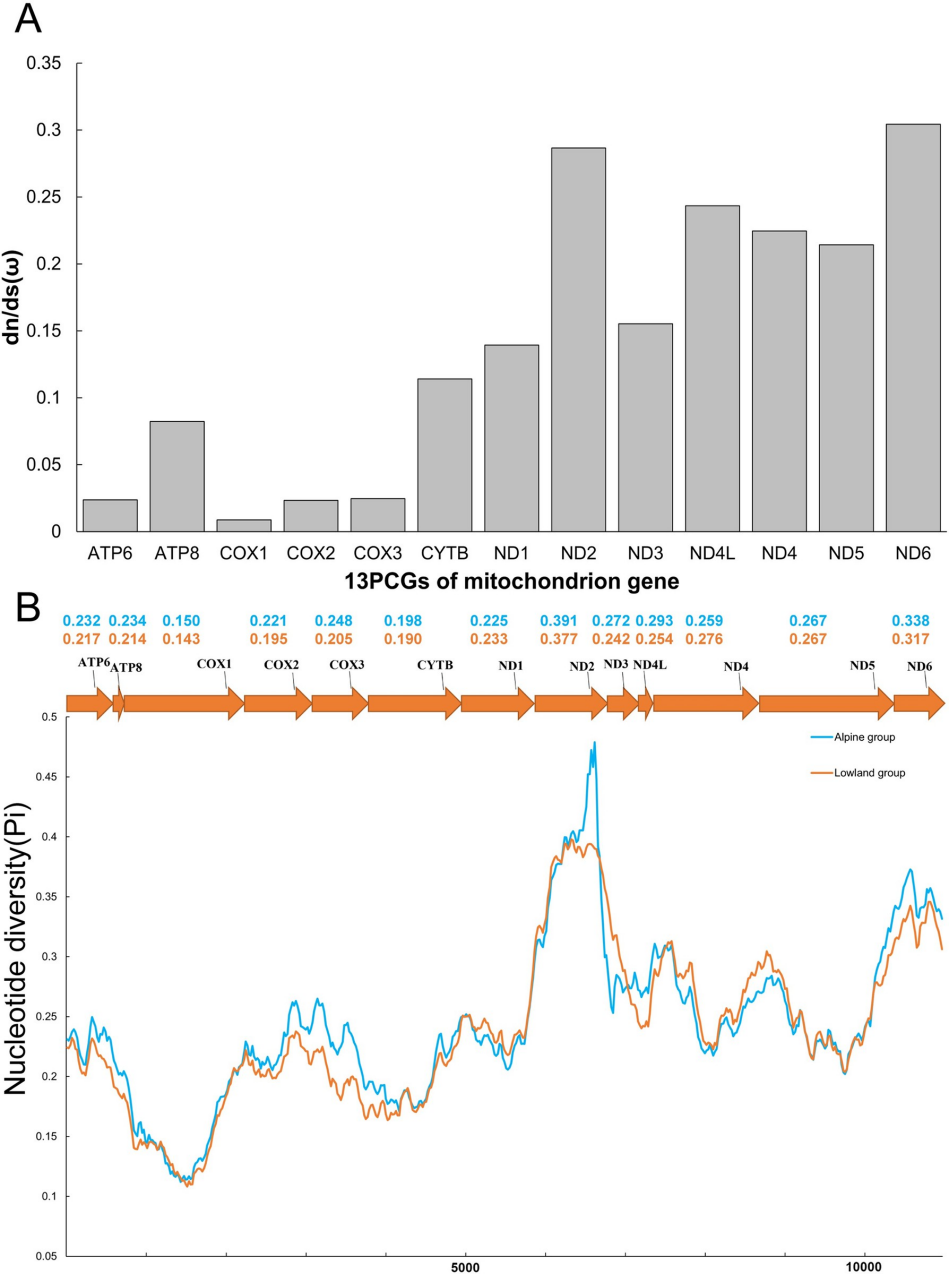
The dn/ds ratio and nucleotide diversity of 13 PCGs in 29 sequences of Pteromalidae and Eulophidae A. The dn/ds ratio of each PCGs. B. The nucleotide diversity (Pi) of 13 PCGs. Line showed the sliding window analysis results. The blue line indicates the nucleotide diversity of the alpine group and the yellow line shows the nucleotide diversity of lowland group. The values of corresponding color represent the nucleotide diversity.

Their ω value showed completely different trend, *COX1*(0.009) showed the lowest evolutionary rate, while the ω value of *NADH* complex enzyme (mean 0.22) was significantly higher than that of other genes. At the same time, the nucleotide diversity in 13 PCGs also differed among groups and genes (Fig 3). The nucleotide diversity value was slightly higher in the alpine group, with *COX1* (Pi = 0.15) showing the lowest polymorphism and *ND2* (Pi = 0.391) exhibiting the highest. In the lowland group, nucleotide diversity was similarly lowest in *COX1* (Pi = 0.143) and highest in *ND2* (Pi = 0.377), while there was no significant difference between them.

**Fig 4:**
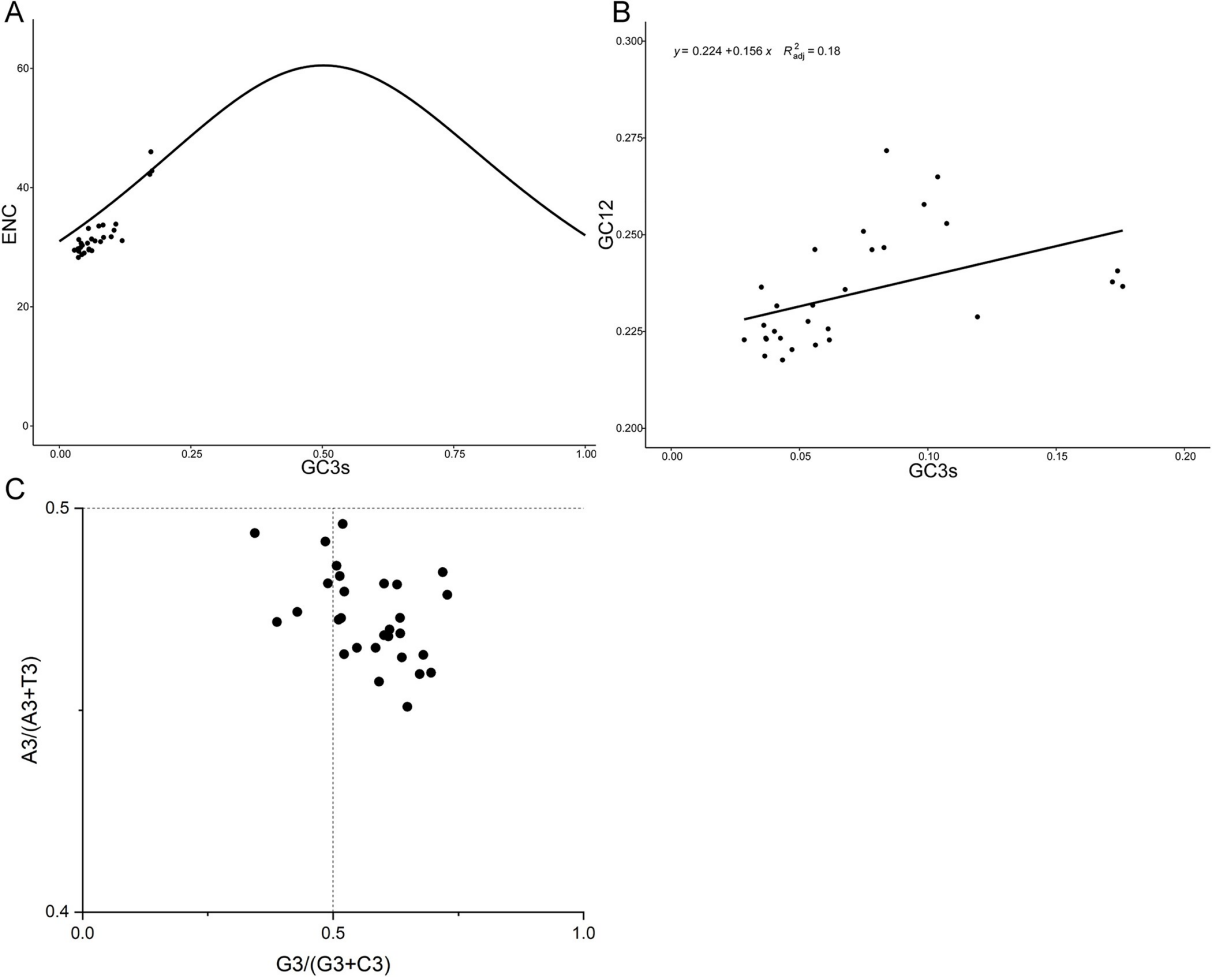
Codon analysis results of 13 PCGs among 29 species. (A)The ENC-GC3s plot. (B) The neutrality plots. (C)The PR2 bias plot. Each point represents a species.

To better understand the dominant factors driving the codon usage patterns of mitochondrial genes and the related determinants, we subsequently performed ENC-GC3s plot, neutrality plot, and PR2 analysis (Fig 4). The ENC-GC3s plot showed that ENC value ranged from 28.3 (*Asaphes vulgaris*) to 46 (*Trichomalopsis sarcophagae*), and the average value of 32.1 was significantly lower than 35, indicating an obvious codon preference (S2 Table in S1 File, Fig 4A). Notably, most species are below and distant from the expected curve, indicating that natural selection is likely a more dominant influence on codon usage preference than mutation. The GC3s are relatively narrow, ranging from 0.028 to 0.176, and the correlation with GC12 was not significant (Fig 4B). The slope of the regression line of the neutrality plot indicated the balance between selection and mutation, the correlation between GC3s and GC12 was relatively low (R^2^ = 0.18), with the regression line was nearly parallel to the X-axis, suggesting that a more pronounced role of selection compared to mutation. The PR2 analysis illustrated the relationship between A and T, C and G at the codon’s third base (Fig 4C). Most data points were clustered in the lower right, the lopsided distribution pattern suggests these species might be affected by other factors like base mutation and translational selection.

### Phylogenetic analysis

**Fig 5:**
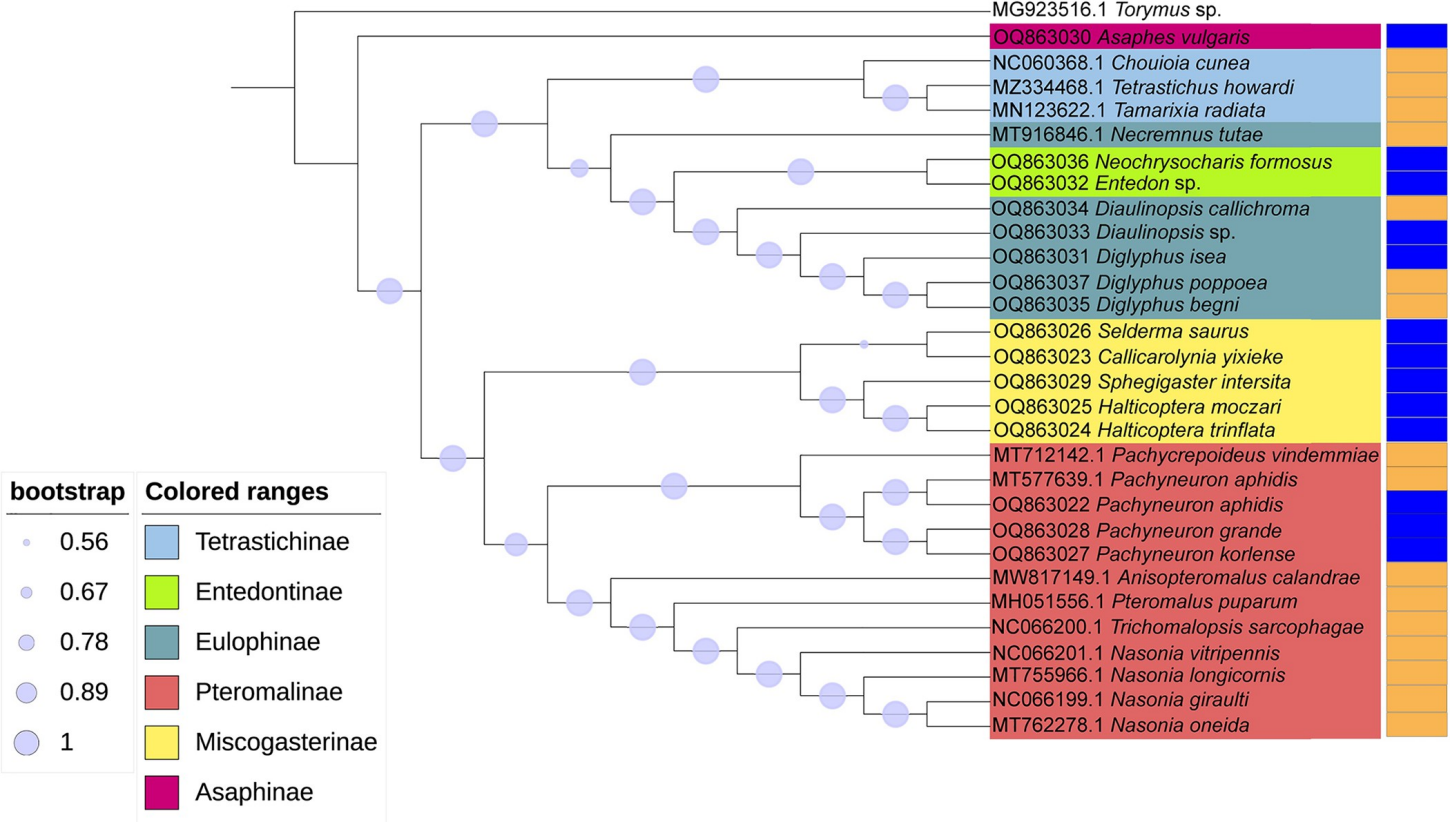
BI phylogenetic tree of all 29 species analyzed in this research based on 13 PCGs mtDNA. The background colors of species’ name indicated different subfamilies. The blue color represents the alpine group and the orange color represents the lowland group.

The species of Pteromalidae and Eulophidae belonging to the same subfamilies were mostly clustered in a well-supported clade with high bootstrap value and Bayesian posterior probabilities (Fig 5). Species from the same genus also clustered together. These phylogenetic relationships provide favorable support to traditional morphological identification, which was also consistent with previous researches [10, 42].

### Selective pressure analysis

The ω value <1, = 1, or >1 indicates purifying (negative) selection, neutral selection, and diversifying (positive) selection, respectively. Neither the site model nor the branch model identified any sites under positive selection, suggesting that purifying selection might be the predominant force driving mtDNA alpine adaptive evolution. The results of the branch model indicated that the highest ω value was observed in *ND2* (0.295), while *COX1* (0.199) showed the lowest ω value, the most conserved gene *COX1* is widely used for species delimitation [43]. The two-ratio model showed that the ω value of eight genes (*ATP6*, p = 0.157; *ATP8*, p = 0.138; *COX2*, p = 0.368; *CYTB*, p = 0.032; *ND1*, p = 0.464; *ND2*, p = 0.913; *ND3*, p = 0.112; *ND4L*, p = 0.867) was higher in the alpine group than in the lowland group (S3 Table in S1 File). We then analyzed the signals of positive selection with the MEME, FEL, FUBAR, and SLAC models. For all the genes with positive selection sites identified by the MEME model, FEL detected 9 positively selected codons in six genes (*ATP6*, *ATP8*, *COX3*, *ND2*, *ND5*, *ND6*) (Table 2), three codons in two genes (*ATP6* and *COX3*) using FUBAR and one codon in *CYTB* using SLAC. The episodic diversifying selection sites were significantly less than the purifying selection sites. The results indicated that Pteromalids and Eulophids from different altitudes were primarily evolving under purifying selection, accumulating non-synonymous mutations in specific mitochondrial PCGs.

**Table 2:** Codons that candidates for experiencing positive selection through four selection analyses.

Gene	MEME (p<0.05)	FEL (p<0.1)		FUBAR (pp>0.9)			SLAC (p<0.1)	
		Positive	Negative	Positive		Negative	Positive	Negative
*ATP6*	5	1	151	2	168		0	117
*ATP8*	1	1	21	0	17		0	8
*COX1*	6	0	375	0	456		0	336
*COX2*	3	0	158	0	172		0	118
*COX3*	10	1	183	1	213		0	136
*ND1*	8	0	219	0	235		0	159
*ND2*	7	3	193	0	221		0	117
*ND3*	1	0	81	0	87		0	58
*ND4*	11	0	265	0	311		0	175
*ND4L*	0	0	58	0	54		0	34
*ND5*	7	1	335	0	405		0	247
*ND6*	6	2	109	0	114		0	58
*CYTB*	4	0	289	0	312		1	233

Finally, the branch-site model was used to detect positive selection sites for the alpine and lowland group through CodeML in PAML. The results identified seven sites in five genes (*ATP6*, *ATP8*, *COX1*, *COX3*, and *CYTB*) when the branches from alpine were set as foreground lineage, and the genes under positive selection most in the alpine parasitoids (S4 Table in S1 File). According to branch-site model analysis, the above genes with positive selection signals have higher evolution rates in the alpine group (ω>1), *Calicarolynia yixieke*, *Asaphes vulgaris*, and *Sphegigaster intersita* branches with higher substitution rates for all five genes.

### Phylogenetic independent contrast and environmental analysis

**Fig 6:**
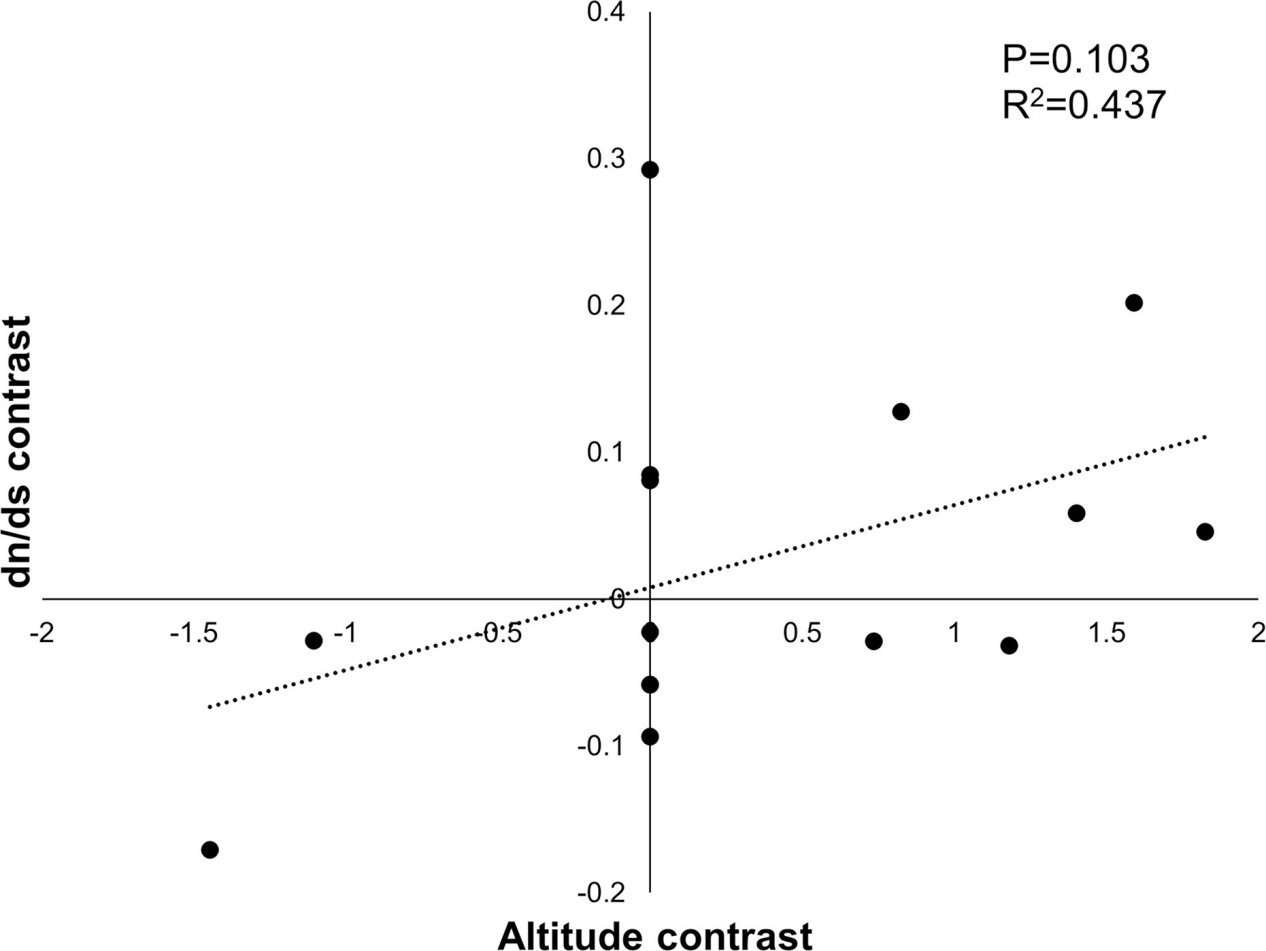
The PIC analysis of between different altitude and dn/ds ratio of 13 mtDNA PCGs.

We used PIC analysis to avoid the interference of evolutionary information. The results showed an increasing trend in the dn/ds ratio with increasing altitude (Fig 6), which also verified that the evolution rate of 13 mtDNA PCGs was relatively higher in the alpine group when compared with the lowland group.

The Mantel test results showed a significant correlation between the evolution rate and these environmental factors (r = 0.905, p = 1×10^−4^). The generalized linear models’ results indicated that the evolution rate of four genes (*ATP6*, *ATP8*, *COX3*, and *ND1*) had a significant relationship with the temperature-related environmental factors (S5 Table in S1 File). The dn/ds ratio of *ATP6*, *COX3*, and *ND1* was correlated with the minimum temperature of the coldest month (BIO6, p = 4.72×10^−5^), mean temperature of the coldest quarter (BIO11, p = 0.04), and mean temperature of the driest quarter (BIO9, p = 0.015), respectively. A significant correlation was observed between the dn/ds ratio of *ATP8* and temperature seasonality (BIO4, p = 0.04), mean temperature of the warmest quarter (BIO10, p = 0.04), and mean temperature of the coldest quarter (BIO11, p = 0.04). Other precipitation-related factors were excluded through stepwise regression.

## Discussion

Mitochondrial genes have been widely used in studies of environmental adaptation in various alpine organisms [19]. In this study, 16 mitogenomes of Pteromalidae and Eulophidae from plateau and basin deserts were sequenced, then a comparative analysis of these mitogenomes’ evolution was conducted. The results of codon usage preference analysis suggest that these species might be affected by natural selection. We found positive selection signals in five PCGs (*ATP6*, *ATP8*, *COX1*, *COX3*, and *CYTB*) at high altitude branches, and most genes are more likely under relaxed purifying selection with their evolution rate less than 1.

The general features of mitogenomes were relatively conserved in the sequenced species, except that trnM, V, A, Q, and I were missing to varying degrees, the incomplete mitochondrial genomes, including missing tRNA, PCGs, and AT-rich region, are relatively common in Hymenoptera [7, 44–46], *Encarsia obtusiclava* (Chalcidoidea: Aphelinidae) lacks *trnV* and *tnrM* [44], and 18 species of Chalcidoidea showed varying degrees of tRNA and PCGs deletions [10]. The lost mitochondrial genes may have been functionally transferred to the nucleus as mitochondrial pseudogenes, a general phenomenon in various insects [47]. This phenomenon warrants further verification through in-depth sequencing in additional similar species.

The AT content of Hymenoptera insects is usually higher than that of other insects [48]. In our study, the AT content of Pteromalid and Eulophids’ PCGs ranges from 78.6% to 83.8% (Table 1, S1 Fig in S1 File), which also makes it more difficult to obtain complete genome sequences. The RSCU value could directly reflect the usage rate of synonymous codons. Most of the preferred codons ended with A/T base, such a preference might be caused by the AT-rich sequence composition. The codon bias in organisms or genes is influenced by the G+C content, natural selection, and mutation [14]. The ENC value ranges from 28 in *Asaphes vulgaris* to 46 in *Trichomalopsis sarcophagae* (mean = 32) (S2 Table in S1 File) indicating significant codon preference and there might be mutational pressure on relative species [49], the slope of the neutrality plot regression line closer to zero suggests that natural selection is the dominant driver of codon usage bias. The PR2 analysis results showed that most of the points were located in the lower right part, indicating that the codon bias might be caused by mutations [50]. The synonymous mutation sites in the PCGs are highly correlated with the gene length and nonsynonymous sites, suggesting that they might evolve in a neutral pattern.

The 13 PCGs in the mitogenome are the crucial components of ATP synthase, cytochrome c oxidase, *NADH* dehydrogenase, and coenzyme Q, all of which are responsible for energy and heat production. Combined with the results of evolution rate and nucleotide polymorphism, it is speculated that cytochrome c oxidase and *NADH* dehydrogenase may be subjected to more selection pressure. The results of dn/ds ratio showed that the ω value of 13 PCGs was all less than 1, indicating that those genes were under purifying selection to a larger extent (Fig 3). Based on the BI phylogenetic relationships of those species, the site model, branch model, and branch-site model were conducted. Neither of the two pairs of site models (M1a vs M2a; M7 vs M8) nor the branch model detected any genes or sites under positive selection. The branch model identified that the evolutionary rate of eight genes (*ATP6*, *ATP8*, *COX2*, *CYTB*, *ND1*, *ND2*, *ND3*, *ND4L*) in the alpine group was higher than that in the lowland group (S3 Table in S1 File), indicating that the alpine group has accumulated more nonsynonymous mutation sites during evolution, which is similar to the results of the PIC analysis, showing that the dn/ds ratio increased with elevation. Combined with the results of branch-site model and Hyphy’s model, five positively selected PCGs (*ATP6*, *ATP8*, *COX1*, *COX3*, and *CYTB*) were identified, which resembled the results of selective signals in Tibetan grasshoppers (*ATP6*, *ATP8*, *ND1-5*) (Orthoptera) [21], the signatures of positive selection were detected at codon 404 of *COX1* in Tibetan Lycaenidae (Lepidoptera) [51], *ATP6* and *ND5* showed positively selected signatures in Tibetan *Dolycoris baccarum* (Hemiptera), and genes with positive selection signals found in other Tibetan organisms [19]. The ATP synthase contains two subunits, *ATP6* plays an important role in rotor performance, while *ATP8* is the central part of the F0 component [52], the enzyme is widely distributed in the inner mitochondrial membrane and has an essential role in catalyzing the synthesis of ATP. *CYTB* has catalytic activity and is the only mtDNA-derived subunit of complex Ⅲ. *COX1* and *COX3* are both core parts of complex Ⅳ and are mainly responsible for catalyzing the electron transfer to the ultimate receptor of molecular oxygen. These PCGs with positive selection sites are likely to have evolved to adapt to specific environmental conditions or functional requirements.

Overall, variations in the components of the oxidative phosphorylation pathway are also correlated with environmental adaptations. Mitochondrial DNA mutations and functions are largely influenced by environmental stress including diet and temperature [53, 54]. The results of the generalized linear model analysis combing 19 environmental factors and evolution rate values showed that temperature-related factors have a significant influence on the evolution rate of *ATP6*, *ATP8*, *COX3*, and *ND1*. In conclusion, temperature has a significant effect not only on their morphological size [3] but also on the evolutionary rate of their mitochondrial PCGs. However, their specific response to temperature needs to be further explored.

Due to the extensive communication between mitochondrial and nuclear genes [53], and the strong signals of mitonuclear coevolution [55], a more integrated analysis of mitochondrial and nuclear genes is necessary to further understand the role of parasitoids’ genomic evolution. We also aim to further verify these results with more closely related species to obtain more corroborative results.

## Conclusion

This research examines the relationship between evolutionary rate of mtDNA PCGs, altitude, and environmental factors in Pteromalidae and Eulophidae at high and low altitude regions.

The mitochondrial protein-coding genes showed an obvious AT bias and codon preference in eight codon families (Ser, Leu, Ala, Arg, Gly, Pro, Thr, Val), the most frequently used codons usually ended with A or T, suggesting that natural selection might play a more significant role in codon usage preference than mutation. The nucleotide diversity and evolutionary rate in the alpine group were higher than those in the lowland group. *ATP6*, *ATP8*, *COX1*, *COX3*, and *CYTB* showed seven positive selection sites, while *ATP6*, *ATP8*, *COX3*, and *ND1* were strongly associated with temperature-related environmental factors. The results offer insights into the adaptative mechanisms of parasitoids in response to the alpine environment and consequently illuminate how these species might react to future ambient temperature shifts.

## Supporting information

S1 File (DOCX)
