# Supplementary material for: Adaptive evidence of mitochondrial genes in Pteromalidae and Eulophidae (Hymenoptera: Chalcidoidea)
Source: PLoS One. 2023 Nov 21;18(11):e0294687. doi: 10.1371/journal.pone.0294687 (PMC10662703; doi:10.1371/journal.pone.0294687)
Supplement: S1 File — (DOCX) [file pone.0294687.s001.docx]

**Support information**

**S1 Table** Basic information of the 13 PCGs mtDNA from 29 species analyzed in this study.

| **Family** | **Subfamily** | **Species** | **Accession number** | **Size (bp)** | **A+T (%)** | **Geographical information** |
| --- | --- | --- | --- | --- | --- | --- |
| Pteromalidae | Miscogasterinae | ***Callicarolynia yixieke*** | **OQ863023** | 16025 | 81.5 | N:37.306109, E:90.342572, 3907.8m |
|  |  | ***Halticoptera trinflata*** | **OQ863024** | 13976 | 84.5 | N:36.931421, E:90.281182, 4071.65m |
|  |  | ***Halticoptera moczari*** | **OQ863025** | 16538 | 85.4 | N:36.940516, E:90.280066, 4052.62m |
|  |  | ***Selderma saurus*** | **OQ863026** | 13942 | 90.6 | N:37.803885, E:89.91128, 3502.74m |
|  |  | ***Sphegigaster intersita*** | **OQ863029** | 16151 | 89.7 | N:38.072916, E:89.119638, 3681.55m |
|  | Asaphinae | ***Asaphes vulgaris*** | **OQ863030** | 14171 | 83.5 | N:37.803944, E:89.91133, 3497.52m |
|  | Pteromalinae | ***Pachyneuron aphidis*** | **OQ863022** | 15389 | 85 | N:37.66275, E:89.807278, 4028.22m |
|  |  | ***Pachyneuron korlense*** | **OQ863027** | 15033 | 84.4 | N:38.068995, E:89.03947, 3785.43m |
|  |  | ***Pachyneuron grande*** | **OQ863028** | 15163 | 85.9 | N:38.09378, E:89.2714167, 3471.91m |
|  |  | *Anisopteromalus calandrae* | MW817149 | 15954 | 82.9 | N:28.2333, E:112.20,143m |
|  |  | *Nasonia giraulti* | NC_066199 | 16415 | 82 | Netherlands. -11m |
|  |  | *Nasonia longicornis* | MT755966 | 14925 | 84.3 | Netherlands. -11m |
|  |  | *Nasonia oneida* | MT762278 | 14811 | 83.5 | Netherlands. -11m |
|  |  | *Nasonia vitripennis* | NC_066201 | 22956 | 74.2 | Netherlands. -11m |
|  |  | *Pachycrepoideus vindemmiae* | MT712142 | 14850 | 85.3 | from author’s unit, 119.3m |
|  |  | *Pachyneuron aphidis* | MK577639 | 15137 | 85.1 | Yuxi, Yunnan province. 1800m |
|  |  | *Pteromalus puparum* | MH051556 | 18217 | 84.7 | from author’s unit, 6m, |
|  |  | *Trichomalopsis sarcophagae* | NC_066200 | 15042 | 82.9 | Netherlands. -11m, |
| Eulophidae | Eulophinae | ***Diglyphus isea*** | **OQ863031** | 14178 | 80 | N:37.803944, E:89.91133, 3497.52m |
|  |  | ***Diaulinopsis* sp.** | **OQ863033** | 13759 | 80.5 | N:37.23281, E:90.40914, 3864m |
|  |  | *Diaulinopsis callichroma* | OQ863034 | 14320 | 82.1 | N:44.37383, E:87.88172, 392.56m |
|  |  | *Diglyphus begni* | OQ863035 | 14478 | 75.4 | N:43.767861, E 87.6125, 957.2m |
|  |  | *Diglyphus poppoea* | OQ863037 | 13193 | 79.2 | N:43.767861, E 87.6125, 957.2m |
|  |  | *Necremnus tutae* | MT916846 | 15252 | 84.3 | N:41.05, E:2.36667, 680m |
|  | Entedoninae | *Neochrysocharis formosus* | OQ863036 | 14196 | 88.9 | N:43.767861, E 87.6125, 957.2m |
|  |  | *Entedon* sp. | OQ863032 | 14568 | 84.2 | N:44.731778, E:85.13644, 291.32m |
|  | Tetrastichinae | *Chouioia cunea* | NC_060368 | 14930 | 85.1 | N:110.33583, E:19.989167, 25m |
|  |  | *Tamarixia radiata* | MN123622 | 14752 | 85.4 | N:23.2833, E:116.15, 775m |
|  |  | *Tetrastichus howardi* | MZ334468 | 14791 | 85.5 | N:19.989167, E:110.33583, 16m |

**
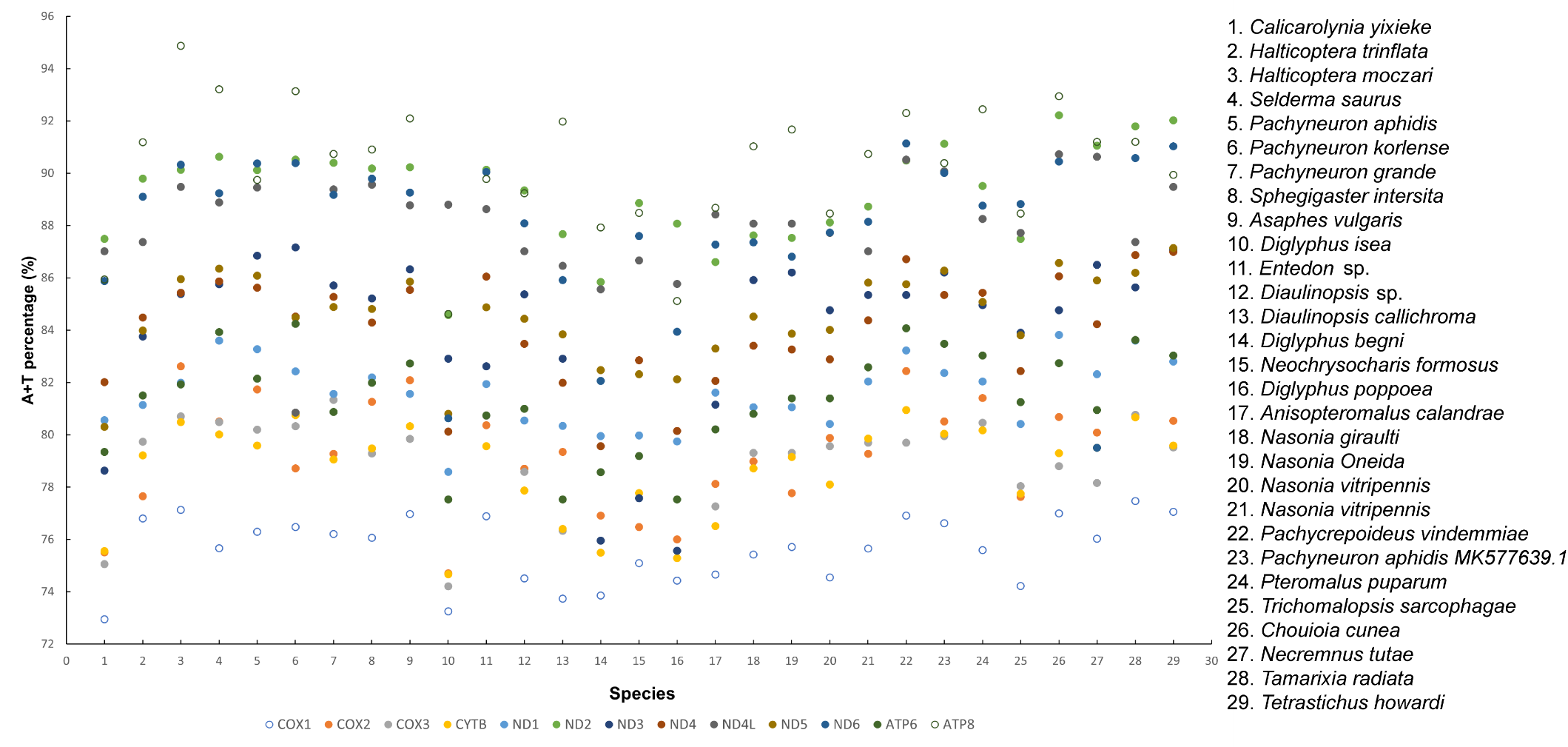
**

**S1** **Fig.** The AT-content of 13 PCGs mtDNA of analyzed 29 Pteromalidae and Eulophidae species.

**S2 Table** The effective number of codons (ENC) and G+C content of the first and second positions (GC12) and the third positions (GC3).

| **Family** | **Subfamily** | **Species** | **ENC** | **GC3** | **GC12** |
| --- | --- | --- | --- | --- | --- |
| Pteromalidae | Miscogasterinae | *Callicarolynia yixieke* | 33.875 | 0.107 | 0.253 |
|  |  | *Halticoptera trinflata* | 31.095 | 0.119 | 0.229 |
|  |  | *Halticoptera moczari* | 28.768 | 0.043 | 0.223 |
|  |  | *Selderma saurus* | 29.302 | 0.037 | 0.223 |
|  |  | *Sphegigaster intersita* | 29.416 | 0.062 | 0.223 |
|  | Asaphinae | *Asaphes vulgaris* | 28.285 | 0.036 | 0.227 |
|  | Pteromalinae | *Pachyneuron aphidis* | 29.019 | 0.047 | 0.220 |
|  |  | *Pachyneuron korlense* | 29.691 | 0.056 | 0.222 |
|  |  | *Pachyneuron grande* | 31.379 | 0.061 | 0.226 |
|  |  | *Anisopteromalus calandrae* | 33.718 | 0.083 | 0.247 |
| Eulophidae |  | *Nasonia giraulti* | 42.81 | 0.176 | 0.237 |
|  |  | *Nasonia longicornis* | 33.157 | 0.055 | 0.232 |
|  |  | *Nasonia oneida* | 31.052 | 0.068 | 0.236 |
|  |  | *Nasonia vitripennis* | 42.225 | 0.172 | 0.238 |
|  |  | *Pachycrepoideus vindemmiae* | 30.318 | 0.043 | 0.218 |
|  |  | *Pachyneuron aphidis* | 29.969 | 0.040 | 0.225 |
|  |  | *Pteromalus puparum* | 30.671 | 0.041 | 0.232 |
|  |  | *Trichomalopsis sarcophagae* | 46.014 | 0.174 | 0.241 |
| Eulophidae | Eulophinae | *Diglyphus isea* | 32.849 | 0.104 | 0.265 |
|  |  | *Diaulinopsis* sp. | 29.553 | 0.056 | 0.246 |
|  |  | *Diaulinopsis callichroma* | 33.553 | 0.075 | 0.251 |
|  |  | *Diglyphus begni* | 31.758 | 0.099 | 0.258 |
|  |  | *Diglyphus poppoea* | 31.647 | 0.084 | 0.272 |
|  |  | *Necremnus tutae* | 29.733 | 0.035 | 0.236 |
|  | Entedoninae | *Neochrysocharis formosus* | 30.683 | 0.053 | 0.228 |
|  |  | *Entedon* sp. | 30.931 | 0.078 | 0.246 |
|  | Tetrastichinae | *Chouioia cunea* | 31.290 | 0.037 | 0.223 |
|  |  | *Tamarixia radiata* | 29.503 | 0.028 | 0.223 |
|  |  | *Tetrastichus howardi* | 29.347 | 0.036 | 0.219 |

**S3 Table** Results of selective pressure analysis (dN/dS ratios) of 13 PCGs in alpine(A) vs. lowland(L) lineages of Pteromalidae and Eulophidae based on one vs. two ratio model.

| Gene | two ratio model dN/dS ratios | | Is A > L dN/dS? | Likelihood value | | P value |
| --- | --- | --- | --- | --- | --- | --- |
|  | Alpine | Lowland |  | One ratio model | Two ratio model |  |
| *ATP6* | 0.0241 | 0.01478 | **TRUE** | -9518.5492 | -9517.4975 | 0.157 |
| *ATP8* | 0.0337 | 0.00662 | **TRUE** | -3298.9766 | -3297.8713 | 0.138 |
| *COX1* | 0.00949 | 0.01065 | FALSE | -14759.2225 | -14758.9665 | 0.4743 |
| *COX2* | 0.01393 | 0.01007 | **TRUE** | -9449.4552 | -9449.0491 | 0.3675 |
| *COX3* | 0.02078 | 0.02286 | FALSE | -11286.1293 | -11286.0536 | 0.6972 |
| *CYTB* | 0.01667 | 0.01099 | **TRUE** | 10976.1722 | 10976.2739 | 0.0315 |
| *ND1* | 0.01151 | 0.00944 | **TRUE** | -12539.2834 | -12539.0154 | 0.4641 |
| *ND2* | 0.00568 | 0.0055 | **TRUE** | -16126.1989 | -16126.1931 | 0.913 |
| *ND3* | 0.01507 | 0.00519 | **TRUE** | -5343.864428 | -5342.654901 | 0.1199 |
| *ND4* | 0.01129 | 0.01344 | FALSE | -20937.8254 | -20937.5710 | 0.476 |
| *ND4L* | 0.00812 | 0.00762 | **TRUE** | -4347.0797 | -4347.0656 | 0.867 |
| *ND5* | 0.00668 | 0.00923 | FALSE | -25983.1453 | -25982.3044 | 0.195 |
| *ND6* | 0.00157 | 0.00604 | FALSE | -10288.5812 | -10287.7192 | 0.189 |

**S4 Table** Results of positive selection on 13 mtDNA PCGs in Pteromalidae and Eulophidae based on branch-site model analysis.

| **Gene** | **Models** | **Parameter** | | | | | **p-value** | | **Positive sites (BEB analysis)** |
| --- | --- | --- | --- | --- | --- | --- | --- | --- | --- |
|  |  | **Site class** | **0** | **1** | **2a** | **2b** | | **(p<0.05)** |  |
| *ATP6* | Null model | Proportion | 0.79072 | 0.02295 | 0.18107 | 0.00526 | | 2.394×10^-5^ | 141V, 1.000** |
|  |  | Background ω | 0.01675 | 1.00000 | 0.01675 | 1.00000 | |  |  |
|  |  | Foreground ω | 0.01675 | 1.00000 | 0.40000 | 0.40000 | |  |  |
|  | Model A | Proportion | 0.75340 | 0.02198 | 0.21825 | 0.00637 | |  |  |
|  |  | Background ω | 0.01701 | 1.00000 | 0.01701 | 1.00000 | |  |  |
|  |  | Foreground ω | 0.01701 | 1.00000 | 1.00000 | 1.00000 | |  |  |
| *ATP8* | Null model | Proportion | 0.32744 | 0.45222 | 0.09254 | 0.12780 | | 0.6375 | 83N, 0.999* |
|  |  | Background ω | 0.09911 | 1.00000 | 0.09911 | 1.00000 | |  |  |
|  |  | Foreground ω | 0.09911 | 1.00000 | 1.00000 | 1.00000 | |  |  |
|  | Model A | Proportion | 0.35497 | 0.40822 | 0.11014 | 0.12667 | |  |  |
|  |  | Background ω | 0.11459 | 1.00000 | 0.11459 | 1.00000 | |  |  |
|  |  | Foreground ω | 0.11459 | 1.00000 | 1.00000 | 1.00000 | |  |  |
| *COX1* | Null model | Proportion | 0.99191 | 0.00809 | 0.00000 | 0.00000 | | 7.8×10^-5^ | 510A, 1.000** |
|  |  | Background ω | 0.01032 | 1.00000 | 0.01032 | 1.00000 | |  |  |
|  |  | Foreground ω | 0.01032 | 1.00000 | 0.40000 | 0.40000 | |  |  |
|  | Model A | Proportion | 0.98856 | 0.00466 | 0.00675 | 0.00003 | |  |  |
|  |  | Background ω | 0.01022 | 1.00000 | 0.01022 | 1.00000 | |  |  |
|  |  | Foreground ω | 0.01022 | 1.00000 | 1.00000 | 1.00000 | |  |  |
| *COX3* | Null model | Proportion | 0.88295 | 0.06507 | 0.04841 | 0.00357 | | 0.0000 | 65Y, 0.992**  182Y, 0.961*  185F, 0.996** |
|  |  | Background ω | 0.02761 | 1.00000 | 0.02761 | 1.00000 | |  |  |
|  |  | Foreground ω | 0.02761 | 1.00000 | 1.00000 | 1.00000 | |  |  |
|  | Model A | Proportion | 0.40343 | 0.03283 | 0.52133 | 0.04242 | |  |  |
|  |  | Background ω | 0.03068 | 1.00000 | 0.03068 | 1.00000 | |  |  |
|  |  | Foreground ω | 0.03068 | 1.00000 | 0.00000 | 0.00000 | |  |  |
| *CYTB* | Null model | Proportion | 0.43668 | 0.01986 | 0.51981 | 0.02364 | | 0.0000 | 275P, 1.000** |
|  |  | Background ω | 0.01736 | 1.00000 | 0.01736 | 1.00000 | |  |  |
|  |  | Foreground ω | 0.01736 | 1.00000 | 0.00000 | 0.00000 | |  |  |
|  | Model A | Proportion | 0.95474 | 0.03808 | 0.00691 | 0.00028 | |  |  |
|  |  | Background ω | 0.01497 | 1.00000 | 0.01497 | 1.00000 | |  |  |
|  |  | Foreground ω | 0.01497 | 1.00000 | 575.669 | 575.669 | |  |  |

| **Genes** | **Environmental factors** | **Coefficient** | **SD** | **P value** |
| --- | --- | --- | --- | --- |
| *ATP6* | BIO6 | -0.032767 | 0.008 | 4.72×10^-5^ |
| *ATP8* | BIO4 | 67.46166 | 27.23420 | 0.0352 |
|  | BIO10 | -73.02321 | 30.62366 | 0.0409 |
|  | BIO11 | 20.26942 | 8.37170 | 0.0385 |
| *COX3* | BIO11 | -0.24884 | 0.10728 | 0.0406 |
| *ND1* | BIO9 | -0.044356 | 0.015680 | 0.0152 |

**S5 Table** The correlation between evolutionary rate of mitochondrial PCGs and environmental factors.

Note: BIO4, temperature seasonality; BIO6, min temperature of coldest month; BIO9, mean temperature of driest quarter; BIO10, mean temperature of warmest quarter; BIO11, mean temperature of coldest quarter.
